# Mapping the Antibody Repertoires in Ferrets with Repeated Influenza A/H3 Infections: Is Original Antigenic Sin Really “Sinful”?

**DOI:** 10.3390/v15020374

**Published:** 2023-01-28

**Authors:** Tal Einav, Martina Kosikova, Peter Radvak, Yuan-Chia Kuo, Hyung Joon Kwon, Hang Xie

**Affiliations:** 1 Basic Sciences Division and Computational Biology Program, Fred Hutchinson Cancer Research Center, Seattle, WA 98109, USA; 2 Laboratory of Respiratory Viral Diseases, Division of Viral Products, Office of Vaccines Research and Review, Center for Biologics Evaluation and Research, United States Food and Drug Administration, Silver Spring, MD 20993, USA

**Keywords:** mapping antibody repertoires, original antigenic sin, immune imprinting, repeated influenza exposures, broadly neutralizing antibody, influenza A H3N2 virus

## Abstract

The influenza-specific antibody repertoire is continuously reshaped by infection and vaccination. The host immune response to contemporary viruses can be redirected to preferentially boost antibodies specific for viruses encountered early in life, a phenomenon called original antigenic sin (OAS) that is suggested to be responsible for diminished vaccine effectiveness after repeated seasonal vaccination. Using a new computational tool called Neutralization Landscapes, we tracked the progression of hemagglutination inhibition antibodies within ferret antisera elicited by repeated influenza A/H3 infections and deciphered the influence of prior exposures on the de novo antibody response to evolved viruses. The results indicate that a broadly neutralizing antibody signature can nevertheless be induced by repeated exposures despite OAS induction. Our study offers a new way to visualize how immune history shapes individual antibodies within a repertoire, which may help to inform future universal influenza vaccine design.

## 1. Introduction

Rapidly evolving pathogens such as influenza frequently change their antigenicity in order to escape the host immune system, and the emergence of antigenically drifted strains necessitates the annual update of seasonal influenza vaccine components. Despite efforts to forecast which strain(s) will be most prevalent, a suboptimal or mismatched vaccine strain may occasionally be selected for vaccine production, resulting in reduced protection [1,2,3,4]. In the US, influenza vaccine effectiveness in the past decades has fluctuated significantly from 10% in the 2004–2005 season [1] to 60% in the 2010–2011 season (https://www.cdc.gov/flu/vaccines-work/effectiveness-studies.htm) (accessed on 11 April 2021) [5]. While vaccine mismatch directly accounts for this low efficacy, pre-existing host immunity also influences vaccine performance [3,6,7,8,9,10,11,12].

An individual’s exposure history, acquired through recurrent infections and/or vaccinations, shapes their unique antibody repertoire and influences their response to newly emerging influenza viruses [6,10,11,12,13,14,15,16,17,18,19]. For example, residual antibodies from prior exposures may grant subsequent protection against viruses with similar antigenicity [15,16,17,20]. However, immune imprinting from viruses encountered early in life can also lead to insufficient de novo antibody response to evolved viruses—a phenomenon called original antigenic sin (OAS) [21]. While the exact mechanisms remain unknown, OAS has been associated with low antibody responses in individuals with repeated seasonal vaccination and has been hypothesized to negatively affect vaccine effectiveness in frequent vaccinees [1,9,20,22,23,24,25,26,27,28]. These reports provide a glimpse of the complex interplay between prior and current immunity, highlighting the influence of immune imprinting that must be addressed in the field of vaccinology. Elucidating the impact of OAS on de novo antibody responses will provide insights for future influenza vaccine development with improved performance.

In this work, we used a newly developed computational tool—Neutralization Landscapes [29]—to track the progression of the hemagglutination inhibition (HAI) responses in ferrets after repeated influenza A/H3 infections, and characterized the HAI antibody patterns induced. By mapping the HAI responses at the single-antibody scale, we demonstrated that repeated influenza A/H3 exposures, despite OAS induction, can expand the breadth of de novo HAI antibody response.

## 2. Materials and Methods

### 2.1. Viruses

The panel of H3N2 viruses used for the study included A/Philippines/2/1982 (Philippines 1982), A/Wisconsin/67/2005 (Wisconsin 2005), A/Uruguay/716/2007 (Uruguay 2007), A/Perth/16/2009 (Perth 2009), A/Victoria/361/2011 (Victoria 2011), A/Texas/50/2012 (Texas 2012), A/Switzerland/9715293/2013 (Switzerland 2013) and A/Hong Kong/4801/2014 (Hong Kong 2014), each of which has served as the prototype for the H3N2 seasonal influenza vaccine component in past decades. All H3N2 viruses were propagated in 9–10-day-old embryonated eggs, and aliquots were stored at −80 °C until use.

### 2.2. Ferret Antisera

Seronegative male ferrets (Triple F Farm) at 15–16 weeks old were infected intranasally at two-week intervals with each of the four H3N2 viruses (V_1_ = A/Uruguay/716/2007 or Uruguay 2007, V_2_ = A/Texas/50/2012 or Texas 2012, V_3_ = A/Switzerland/9715293/2013 or Switzerland 2013, and V_4_ = A/Hong Kong/4801/2014 or Hong Kong 2014) [6]. After ferrets were anesthetized, approximately 10^5^ focus-forming units of virus in a total of 1 mL was delivered into both nostrils per ferret at 0.5 mL per nostril [6]. Ferrets were bled via venipuncture of the cranial vena cava under anesthesia at 14 days after each infection. Sera from four ferrets in each infection scheme were collected for HAI titer determination. All procedures were carried out in accordance with a protocol approved by the Institutional Animal Care and Use Committee of the Center for Biologics Evaluation and Research, US Food and Drug Administration.

### 2.3. HAI Assay

Following pre-treatment with a receptor-destroying enzyme (Denka-Seiken), individual ferret sera were 2-fold serially diluted and were 1:1 (*v*/*v*) incubated with testing virus solution containing 4 hemagglutinin (HA) units per 25 μL at room temperature for 30 min before the addition of 50 μL of 0.75% guinea pig erythrocytes in the presence of 20 nM oseltamivir, as previously described [6,14]. Wells containing PBS only or virus only served as the negative and positive controls in each HAI assay performed. The endpoint HAI titer was defined as the reciprocal of the highest serum dilution that yielded a complete HA inhibition, and a titer 5 was assigned if no inhibition was observed at the starting 1:10 serum dilution. HAI geometric mean titers (GMTs) were calculated, along with the 95% confidence intervals.

## 3. Neutralization Landscapes and Decomposition of Ferret Antisera

Neutralization Landscapes uses monoclonal antibody data to quantify viruses cross-reactivity and enumerate the space of potential antibody inhibition profiles [29]. A landscape is a low-dimensional map where antibodies and viruses are represented as points and antibody-virus distance translates into experimentally measurable neutralization. The positions of eight H3N2 viruses were determined using neutralizing titers from six human monoclonal antibodies targeting the head of influenza HA [29]. Four antibodies (CH65 [30], 5J8 [31], C05 [32], and F045-092 [33]) target the receptor binding site, another antibody (F005-126) cross-links between two monomers within a hemagglutinin trimer [34], and the last binds to hemagglutinin’s lateral patch [35]. Previous serum-based efforts using antigenic cartography suggest that these antibodies do not need to bind to the same epitope [36], and that the landscape can include H1N1- and H3N2-specific antibodies [29]. Moreover, we posit that this landscape will become more accurate as more antibodies targeting different epitopes are added. These assertions can be directly tested by assessing the landscape’s ability to predict unmeasured interactions.

The 50% inhibitory concentrations (IC_50_s) were determined between each antibody and virus pair, and their positions on the neutralization landscapes were fixed using two-dimensional (2D) scaling on the log_10_(IC_50_) values, where an antibody–virus distance of *d* unit translates into IC_50_ = 2*^d^*·10^−10^ Molar [29]. To accommodate the HAI titers used in this study, a few cosmetic changes were made in computation. First, the spacing between gridlines was decreased by a factor of log_10_(½) = 3.3 so that 1 grid unit represents a two-fold decrease in neutralization. Second, HAI titers were converted into absolute Molar units by scanning across different conversion factors and minimizing the absolute mean error between log_2_(measured HAI titers) and log_2_(inferred HAI titers from decomposition). The optimal conversion factor translated an antibody–virus map distance of *d* units into an HAI titer = 6000/2*^d^*. This global conversion factor had been applied to all antisera analyzed in this work.

We decomposed each antiserum by determining which set of antibody coordinates and stoichiometries best matched the measured HAI titers against the eight viruses in the panel. Decomposition proceeded by considering *n* = 1, 2, 3… antibodies until the error of the decomposition decreased below a set threshold [29]. To prevent overfitting, decomposition with an additional antibody was only accepted if it decreased the mean fold-error between measured and inferred titers by ≥20% [29]. The relative fractions of each antibody in the mixture were allowed to vary, although each antibody must comprise ≥10% of the mixture and the sum of all fractions must sum to 100%.

In any neutralization landscape, the abundance of each antibody in a mixture was depicted by the size of the gray circle surrounding it. For any virus lying within a gray circle, the antibody at the center of that circle was predicted to have an HAI titer ≥ 80 against it. If an antibody comprised a fraction *f* of a mixture, then its ability to inhibit a virus decreased by *f*-fold. For example, a monoclonal antibody was surrounded by a circle of radius $d_{\mathrm{mAb}}$ = 6.2 grid units (6000/$2^{d_{\mathrm{mAb}}}$ = 80), while an antibody that comprised a fraction *f* of the serum would be surrounded by a circle of radius $r_{\mathrm{mAb}}-\log_{2}\left(\frac{1}{f}\right)$. When multiple antibodies were present in a serum, their collective inhibition or neutralization against a virus was, thus, computed using a competitive binding model,

IC_50,Competitive_ = (∑*_j_ f_j_*/IC_50_^(*j*)^)^−1^,

where *f_j_* represented the fractional composition of the *j*th antibody and IC_50_^(*j*)^ denoted the concentration at which this monoclonal antibody would neutralize the virus by 50% [29]. In this way, any combination of points (which determined IC_50_^(*j*)^ through antibody–virus map distance) at any stoichiometry (*f_j_*) was translated into the mixture’s collective HAI titer against the virus panel.

## 4. Results

### 4.1. Validation of Neutralization Landscapes

Neutralization Landscapes uses fixed virus coordinates to depict the location and magnitude of constituent neutralizing antibodies within an antiserum elicited by natural infection or vaccination [29]. A key assumption of Neutralization Landscapes is that the neutralization profiles of all dominant antibodies within a serum can be represented as individual points on the map, where the Euclidean distance *d* between each antibody coordinate and virus coordinate translates into an HAI titer of 6000/2*^d^*. This forms a basis set of individual antibody behaviors from which we can determine the minimal combination of antibodies that can replicate a serum’s measurements. This process was previously validated by decomposing mixtures of 2–3 antibodies [29]; here, we extend this work to analyze ferret sera for the first time.

The resulting decompositions predict the functional behavior of the dominant antibodies within serum while neglecting the weaker or less frequent antibodies that do not affect a serum’s HAI profile. Since sera may contain multiple functionally similar antibodies, we call the resulting HAI profiles “antibody signatures*”* as they may represent one or multiple antibodies within the response.

On the neutralization landscapes, the gray regions surrounding each antibody signature indicate an HAI titer ≥ 80 against any virus lying within (we refer to this virus as strongly inhibited by the mixture), and stronger antibody signatures could overwhelm the inhibition of weaker antibodies placed further out on the map.

**Figure 1:**
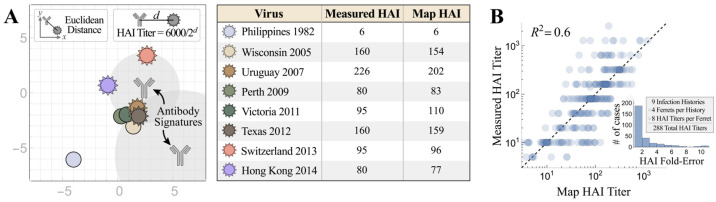
Characterizing the accuracy of Neutralization Landscapes. (**A**) The decomposition of ferret antiserum elicited by sequential H3N2 infections in a neutralization landscape (left) and the resulting predicted/measured HAI titers (right). Larger antibody–virus distance on the landscape corresponds to weaker antibody inhibition, with Euclidean distance *d* representing an HAI titer of 6000/2*^d^*. The resulting antibody signatures represent the predicted inhibition profiles of the dominant antibodies within the serum. (**B**) Cumulative analysis for all ferret antisera analyzed in this work. The inset shows the distribution of fold-errors for these predictions.

To decompose each antiserum, we first determined which combination of coordinates and stoichiometries best matched the experimental HAI titers against the eight H3N2 viruses whose coordinates had been previously fixed using a different antibody–virus panel [37]. The results were validated in two ways. First, Figure 1A showed the decomposition of a ferret antiserum elicited by sequential influenza A/H3 infections in a neutralization landscape in which the HAI titers predicted were on average ≤2-fold off from the experimental measurements (Figure 1A). The same analysis was extended to include all 288 HAI measurements involved in this study, with each point representing a pair of predicted titers and corresponding experimental values in Figure 1B. It yielded a coefficient of R^2^ = 0.6 (Figure 1B), with only 2% (6/288) of map predicted titers being ≥10-fold off from the experimental measurements.

**Figure 2:**
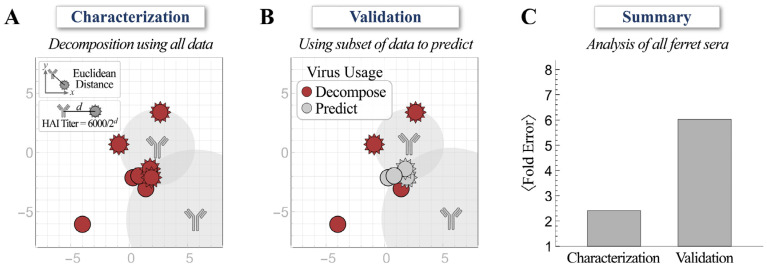
Validating decompositions using a subset of hemagglutination inhibition (HAI) measurements. (**A**) The decomposition of ferret antiserum using experimental HAI titers against the full set of viruses. (**B**) Decomposition of ferret antiserum using experimental HAI titers against the half set of viruses (red) to predict the titers of the remaining four viruses (gray). (**C**) The average error from both methods across all ferret antisera analyzed in this work.

Second, while we decomposed ferret antiserum using the HAI titers against all eight viruses (Figure 2A), we also performed another decomposition by using the half set of measurements to predict the HAI titers against the remaining four viruses in the panel (Figure 2B; titers against red viruses were used for direct decomposition and titers against gray viruses were inferred from the map). Despite the difficulties of triangulating the coordinates and stoichiometries of multiple antibodies using a half set of the measurements, the predicted HAI titers were on average 6.0-fold off from the experimental measurements, only slightly larger than the 2.4-fold error when the full suite of titers was used for decomposition (Figure 2C).

Taken together, these validations demonstrated that the neutralization landscapes accurately characterize the HAI profiles of ferret antisera.

### 4.2. Progression of HAI Responses Following Sequential Infections

**Figure 3:**
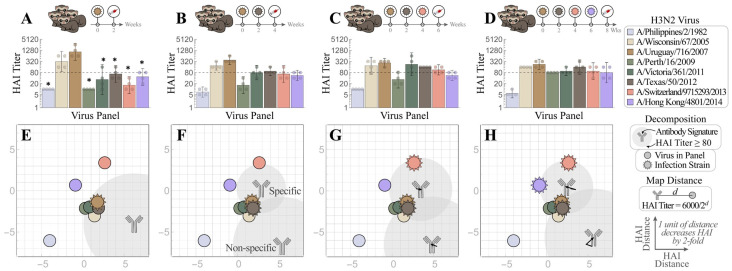
Tracking individual antibodies using hemagglutination inhibition (HAI) responses in ferrets during each stage of four sequential H3N2 infections. (**A**–**D**) Naïve ferrets were sequentially infected with V_1_ = Uruguay 2007 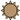, V_2_ = Texas 2012 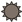, V_3_ = Switzerland 2013 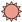, and V_4_ = Hong Kong 2014 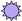. HAI titers are shown for each step of the infection: (**A**) V_1_, (**B**) V_1_→V_2_, (**C**) V_1_→V_2_→V_3_, and (**D**) V_1_→V_2_→V_3_→V_4_. Individual HAI titers are presented from four ferrets (points) with geometric means (bar graphs) and 95% confidential intervals (error bars). * indicates *p* < 0.05 vs. Uruguay 2007 by Mann–Whitney test after data were log transformed. (**E**–**H**) These measurements were decomposed to determine the antibody signatures (the HAI profiles of the dominant antibodies) elicited after infection with (**E**) V_1_ followed by (**F**) V_2_, (**G**) V_3_, and (**H**) V_4_. Each antibody signature (gray) is predicted to have an HAI titer ≥ 80 against any virus within the gray circle, with the size of this circle proportional to the fractional composition of the antibody signature within the serum. An antibody–virus distance *d* denotes an HAI titer of 6000/2*^d^*. Virus coordinates were previously determined using a panel of monoclonal antibodies (see details in the main text). In Panels (**F**–**H**), infection with multiple viruses elicited both specific (HAI ≥ 80 against all infection strains) and non-specific antibody signatures. Arrows show the subtle shifts of the antibody signatures between Panels (**F**)→(**G**)→(**H**).

**Figure 4:**
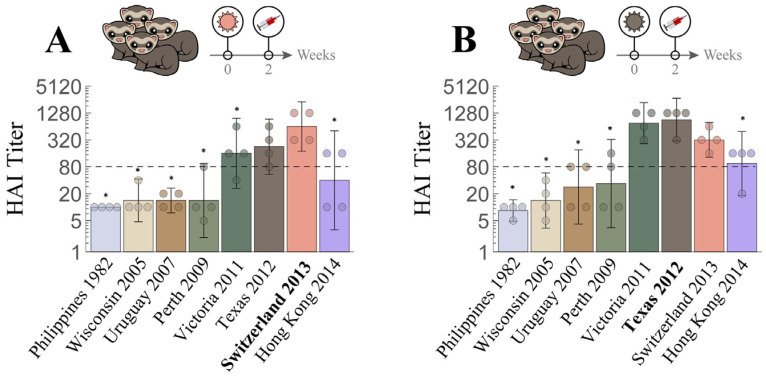
Cross-reactive hemagglutination inhibition (HAI) responses in ferrets with single H3N2 infection. Naïve ferrets were singly infected with either (**A**) V_3_ = Switzerland 2013 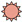 or (**B**) V_2_ = Texas 2012 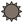. Individual HAI titers from four ferrets (points) and geometric means (bar graphs) are shown with 95% confidential intervals (error bars). * indicates *p* < 0.05 vs. Switzerland 2013 (**A**) or vs. Texas 2012 (**B**) by Mann–Whitney test after data were log transformed.

We first conducted a sequential infection experiment to demonstrate how the antibody repertoire in ferrets was shaped by recurring exposure. Figure 3A–D is the traditional way to present the neutralizing activities of HAI antibodies developed in ferrets after infection with first virus A/Uruguay/716/2007 (Uruguay 2007, denoted as V_1_ throughout this work), then the second virus A/Texas/50/2012 (Texas 2012, V2) (V_1_→V_2_), third A/Switzerland/9715293/2013 (Switzerland 2013, V_3_) (V_1_→V_2_→V_3_), and fourth A/Hong Kong/4801/2014 (Hong Kong 2014, V_4_) (V_1_→V_2_→V_3_→V_4_). Uruguay 2007 (V_1_) infection elicited V_1_-specific ferret HAI titers with limited cross-reactivity towards viruses that emerged before 2005 or after 2007. Following each sequential infection with V_2_, V_3_, and V_4_, the resulting ferret antisera gradually extended the HAI cross-reactivity from V_1_-specific to inhibit (with geometric mean titers (GMTs) ≥ 80) all A/H3 viruses in the panel except A/Philippines/2/1982 (Philippines 1982), which had disappeared from circulation more than three decades earlier (Figure 3B–D). Sequential infections also induced typical OAS, where ferret antisera always had lower HAI titers toward later exposed V_2_, V_3_ or V_4_ than first encountered V_1_ (Supplementary Figure S1A–C). In contrast, infection by V_2_, V_3_ or V_4_ alone (without a priming V_1_ infection) elicited higher homologous HAI GMTs (Figure 4 and Supplementary Figure S1D–F).

We then used Neutralization Landscapes to track the progression of HAI antibodies developed throughout these four infections (Figure 3E–H). Infection by V_1_ showed one antibody signature that strongly inhibited V_1_ and three nearby viruses—A/Wisconsin/67/2005 (Wisconsin 2005), A/Victoria/361/2011 (Victoria 2011), and Texas 2012 (note that Hong Kong 2014 also had a measured titer ≈ 80 (Figure 3A), although this is not seen on the landscape (Figure 3E)). In each subsequent infection (V_1_→V_2_, V_1_→V_2_→V_3_, and V_1_→V_2_→V_3_→V_4_), we detected two distinct antibody signatures; one “specific” antibody (i.e., that strongly inhibited all infection strains) and another “non-specific” antibody signature (that inhibited little-to-no infection strains).

With each additional infection, the specific antibody signature slightly moved by 1–2 units or increased its relative abundance in the mixture (shown by the size of the gray circular regions surrounding each antibody, Figure 3E–H) in a manner that kept all infection strains strongly inhibited. These antibody signatures represent the dominant inhibition profiles within each serum: weaker antibody signatures (including those elicited by earlier infections) may either be masked by these dominant profiles or the profiles of functionally similar antibodies may be combined within one antibody signature. In a sense, these landscapes provide an “Occam’s razor” description of each serum using the minimum possible number of HAI profiles. Note that these landscapes do not perfectly reproduce all titers, but they are highly consistent on average (Figure 1).

Despite OAS induction, ferret antisera after the V_1_→V_2_→V_3_→V_4_ infections had all GMTs within four-fold of one another across the entire virus panel (except for the older Philippines 1982 strain), indicating extended cross-reactivity (Figure 1B–D). The individual maps of Ferrets #1–4 in this cohort also showed similar antibody patterns, i.e., that the end antisera after the V_1_→V_2_→V_3_→V_4_ infections cross-reacted with most viruses in the panel except Philippines 1982 (individual ferret traces shown in Supplementary Figures S2–S4). These progressional maps collectively suggest that a broadly neutralizing antibody signature (defined as an antibody with HAI titer ≥ 80 against multiple infection strains) can be guided into place by sequential exposures despite OAS induction.

### 4.3. Influence of Prior Influenza Exposures on De Novo Antibody Response

**Figure 5:**
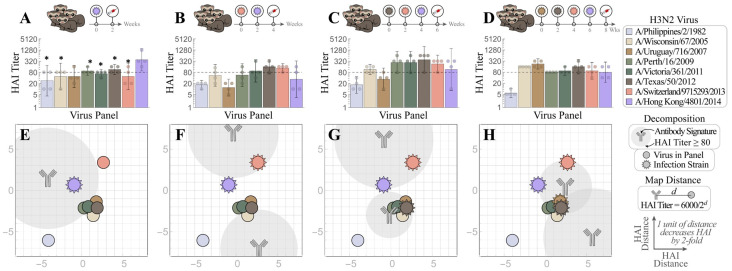
Mapping how exposure history shapes the ferret antibody response. Naïve ferrets were infected with (**A**) V_4_ = Hong Kong 2014 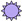, or with prior exposure to (**B**) V_3_ = Switzerland 2013 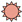, (**C**) V_2_ = Texas 2012 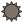, and (**D**) V_1_ = Uruguay 2007 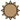. All antisera were analyzed via HAI after the final infection with Hong Kong 2014. Individual HAI titers are shown from four ferrets (points) and geometric means (bar graphs) with 95% confidential intervals (error bars). * indicates *p* < 0.05 vs. Hong Kong 2014 by Mann–Whitney test after data were log transformed. (**E**–**H**) Each set of measurements was decomposed to determine the antibody signatures (the HAI profiles of the dominant antibodies within these sera). Each antibody signature (gray) is predicted to have an HAI titer ≥ 80 against any virus within the gray circle, with the size of this circle proportional to the fractional composition of the antibody signature within the serum. An antibody–virus distance *d* denotes an HAI titer of 6000/2*^d^*.

We next compared the HAI response of ferrets infected with V_4_ alone with the responses elicited after one (V_3_→V_4_), two (V_2_→V_3_→V_4_), or three prior infections (V_1_→V_2_→V_3_→V_4_), to assess how exposure history affected the de novo HAI antibody response to the latest infection by V_4_. Unlike infection by V_4_ alone, which elicited higher HAI titers toward itself than to most viruses in the panel (Figure 5A), ferret antisera induced after additional prior exposures had HAI GMTs toward V_4_ not higher than those toward the earlier infection strains (Figure 5B–D), a typical OAS response that was also seen for every sequence of infections in Figure 3B–D.

We then used Neutralization Landscapes to discern how prior exposures impacted subsequent antibody development at the single-antibody scale (average response shown in Figure 5E–H and individual responses shown in Figure S2). Upon infection by V_4_ alone, one specific antibody signature emerged that strongly inhibited V_4_ (Figure 5E). In fact, a dominant antibody signature specific for the infecting strain was observed in all individual ferrets after single infection (regardless of virus), although an additional minor antibody signature may also be seen in some ferrets (Figure S3).

Ferrets infected by two or more strains consistently showed a polyclonal response, with a specific antibody signature targeting the infection strains and a non-specific antibody signature targeting other viruses in the panel (Figure 3F–H and Figure 5F–H). While multiple infections often resulted in a single predicted antibody signature that strongly inhibits all infection strains (Figure 5F,H), one triple infection resulted in a broad response mediated by two distinct antibody signatures (Figure 5G: Switzerland 2013 and Hong Kong 2014 were strongly inhibited by the top antibody signature, while Texas 2012 was strongly inhibited by the bottom signature). Multiple infections always resulted in OAS, since any antibody signature that strongly inhibited a prior infection strain exhibited weaker inhibition against V_4_ (i.e., V_4_ lies further from the center of the gray antibody circles than the earlier infection strains, Figure 5F–H). Nevertheless, following the V_1_→V_2_→V_3_→V_4_ infections, ferrets developed antibodies that inhibited not only all four infection strains but also the other H3 viruses in the panel, except Philippines 1982.

Taken together, these results suggest that prior exposures affect the antibody response, but that a broadly neutralizing antibody signature can be induced by repeated exposures, even in the presence of OAS.

## 5. Discussion

It is estimated that most humans are infected with influenza by the age of 3 and continue to be reinfected by antigenically drifted strains every 5–10 years [38,39]. Given the variability in infection histories and the stochastic processes involved in each specific infection, it is exceedingly difficult to determine the composition of pre-existing immunity and how it affects an individual’s antibody repertoire. Traditionally, antigenic cartography recomputes the antigenic positions of viruses based on the inhibition capacity of sera used in each study [3,6,14,40,41]. This does not take into account the relationships between viruses inferred from previous studies and complicates efforts to map the antibody repertoire within individual sera. In contrast, the newly developed Neutralization Landscapes uses fixed virus coordinates (determined by a panel of well-defined monoclonal antibodies) to characterize the polyclonal antibodies of the sera [29].

This framework utilizes serological assays to peer into the antibody response to assess the number and inhibition profiles of the antibodies within. Such questions cannot be directly addressed from serum measurements, yet they are crucial to resolve when polyclonal serum is dominated by a single antibody signature (that can be highly susceptible to virus escape mutants) versus multiple antibodies working together. Moreover, this framework can assess the breadth and potency of the antibodies elicited by each virus exposure, providing a vantage to study how preexisting immunity shapes the subsequent antibody response.

These landscapes emphasize that knowing the cross-reactivity relationships between viruses (i.e., the coordinates of each strain on the landscape) and a ferret’s infection history is insufficient to fully predict the antibody response. Landscapes breaks this degeneracy by using HAI titers to search through the space of antibody phenotypes and describes the antibody signatures within sera.

In this study, we combined Neutralization Landscapes with a ferret reinfection model to dissect the collective HAI antibody responses after repeated infections, and deciphered the influence of prior exposures on de novo antibodies. Using four recent H3N2 vaccine strains—Uruguay 2007 (V_1_) from the 2008 to 2010 seasons, Texas 2012 (V_2_) from 2013 to 2015, Switzerland 2013 (V_3_) from 2015 to 2016, and Hong Kong 2014 (V_4_) from 2016 to 2018—we tracked ferret HAI antibody footprints after each step of the sequential infections V_1_→V_2_→V_3_→V_4_ and mapped de novo HAI responses under four scenarios of prior exposures (V_4_ alone, V_3_→V_4_, V_2_→V_3_→V_4_, and V_1_→V_2_→V_3_→V_4_). After two or more infections, we found that the ferret antibody repertoire often contained at least one “specific” antibody signature that strongly inhibited all infection strains, and at least one “non-specific” antibody signature that weakly interacted with other H3 viruses in the panel. Along each step of the exposures V_1_→V_2_→V_3_→V_4_, both the specific and non-specific signatures tended to move closer to the infection strains. Eventually, a single cross-reactive antibody signature emerged that potently inhibited all four infection strains and the rest of the virus panel except Philippines 1982 (Figure 3H). These results are consistent with the hypothesis that with each additional infection, antibodies are refocused on conserved epitopes or structural regions across different infection strains [42,43,44].

In a classical immune response to the same antigen, the reaction from the primary exposure is greatly magnified in subsequent encounters. OAS occurs upon exposure to antigenically related viruses, where the response is skewed more heavily towards the earlier infection strains than to the latest strain. While this OAS phenomenon has been suggested to decrease vaccine effectiveness [9,18,27,45,46,47], seasonal vaccination can persistently extend the number of strains that the human antibody repertoire potently inhibits even when antibodies against earlier viruses are back-boosted [40]. In this study, we observed OAS with each subsequent infection, regardless of the total number of exposures (Figure 3B–D and Figure 5B–D). Despite the ubiquity of OAS, the cross-reactivity of ferret antisera increased with each additional infection. Previously, we also demonstrated that repeated A/H3 infections enhanced antibody avidity toward both early and later exposed viruses, resulting in extended antibody cross-reactivity, despite the induction of OAS costs de novo antibodies specific for contemporary viruses [6]. A recent study has also reported that ferrets sequentially immunized with antigenically different recombinant H3 HAs develop broadly neutralizing antibody responses and are more resistant to antigenically distinct viruses [48]. In humans, seasonal vaccination can also persistently extend cross-reactive antibody landscapes, especially with antigenically advanced vaccine strains, although antibodies against early exposed viruses are back-boosted by seasonal vaccination as well [40]. It is reported that both OAS and non-OAS antibodies originate from clonally related B cells and target the same general regions of HA, although OAS antibodies bind with low affinities [49]. Understanding the mechanisms that drive clonal selection of non-OAS antibodies with broad cross-reactivity is crucial for next generation universal influenza vaccine development.

In this study we also noticed that the inhibition profiles of ferret antibodies following V_1_→V_2_ or V_3_→V_4_ infection were different, despite both schemes showing OAS. Antibodies derived from V_1_→V_2_ infection strongly inhibited both V_1_ and V_2_ (Figure 3B,F), whereas antibodies generated after V_3_→V_4_ infection produced a strong HAI response only against V_3_ and a weak inhibition against V_4_ (Figure 5B,F). Since the first infection by V_1_ or V_3_ resulted in high homologous neutralizing titers (HAI ≥ 320) in all eight ferrets, we hypothesize that the different outcomes for the subsequent infection are due to the antigenic distance between the first and second infecting strains. In the neutralization landscapes, V_1_ (Uruguay 2007) and V_2_ (Texas 2012) are antigenically similar (within 0.6 antigenic units) and, hence, resemble primary and secondary infections by nearly identical viruses, while V_3_ (Switzerland 2013) and V_4_ (Hong Kong 2014) are separated by 4.6 antigenic units and are analogous to infection by two distinct strains [29]. Further experiments are warranted to verify whether similar antibody responses correlate with virus antigenic distances.

Of note, the Neutralization Landscapes in this study used fixed virus coordinates that were pre-determined by human monoclonal antibodies, which may fundamentally differ from those built on ferret antisera [29,37]. For example, Uruguay 2007 (V_1_) and Texas 2012 (V_2_) are considered antigenically distinct by ferret antisera raised from a single infection (Figure 3A and Figure 4B), whereas these two strains are considered antigenically similar and lie close together on the neutralization landscapes (Supplementary Figure S5) [3]. These antigenic differences between human and ferret antibody-based characterizations may arise because of differences between the HAI and neutralization assays, or because humans have complex immune histories that imprint an antibody repertoire, whereas reference ferret antisera are raised in naïve ferrets exposed to a single virus strain [3]. Moreover, some inconsistencies between the landscape titers and the measurements are expected so to avoid overfitting the intrinsic noise of the HAI assay or the heterogeneous responses between individual ferrets. Nevertheless, the full suite of HAI titers presented on the neutralization landscapes shows an average two-fold error to the experimental measurements (Figure 1B and Figure 2C), demonstrating that the antigenic relationships among the majority of the viruses in the panel are the same across humans and ferrets.

In summary, by tracking the changes in the inhibition profile of ferret antisera induced by repeated influenza A/H3 infections, we demonstrated that a broadly neutralizing antibody could be guided along the map after a series of infections. We further show that prior immune history can heavily influence the ferret antibody repertoire. In ferrets exposed to two or more viruses, a broadly neutralizing antibody signature that potently inhibited all infection strains was, nevertheless, produced at the expense of de novo HAI antibodies (Figure 3H). While our current work was focused on HA head-specific antibodies, it does not consider antibodies directed towards the HA stem and neuraminidase that have also been shown to exhibit OAS and may influence the dynamics of this system [50,51]. Hence, complementing this framework with binding or neutralization landscapes would help to resolve the head- versus stem-directed antibody response. Ongoing work will refine these antibody trajectories across multiple infections and multiple regions of an influenza virus, which will help to develop strategies that further expand the broadly neutralizing antibody pool via vaccination to protect against emerging influenza strains.

## Data Availability

Raw HAI datasets generated in the study are available on request from the corresponding author.

## Supplementary Materials

The following supporting information can be downloaded at: https://www.mdpi.com/article/10.3390/v15020374/s1, Figure S1: Hemagglutination inhibition (HAI) responses in ferrets with sequential H3N2 infections; Figure S2: Tracing individual ferret responses through four sequential infections; Figure S3: Tracing individual ferret responses with four different prior infection histories; Figure S4: Antibody repertoires from single infections of ferrets; Figure S5: Comparing neutralization and HAI virus landscapes.
