# Supplementary material for: Mapping the Antibody Repertoires in Ferrets with Repeated Influenza A/H3 Infections: Is Original Antigenic Sin Really “Sinful”?
Source: Viruses. 2023 Jan 28;15(2):374. doi: 10.3390/v15020374 (PMC9959794; doi:10.3390/v15020374)
Supplement: Supplementary file 1 [file viruses-15-00374-s001.zip › viruses-2169529-supplementary.pdf]

Supplementary Information

Mapping the Antibody Repertoires in Ferrets with Repeated Influenza A/H3 Infections: Is Original Antigenic Sin Really "Sinful"?

Tal Einav[1,*,†], Martina Kosikova[2,†], Peter Radvak[2], Yuan-Chia Kuo[2], Hyung Joon Kwon[2], Hang Xie[2,*]

[1]Basic Sciences Division and Computational Biology Program, Fred Hutchinson Cancer Research Center, Seattle, Washington, United States of America.

[2]Laboratory of Respiratory Viral Diseases, Division of Viral Products, Office of Vaccines Research and Review, Center for Biologics Evaluation and Research, United States Food and Drug Administration, Silver Spring, Maryland, United States of America.

* Correspondence: Tal Einav (Email: teinav@fredhutch.org) & Hang Xie (Email: hang.xie@fda.hhs.gov).
† These authors contributed equally to this work.

Five supplementary figures in the following pages

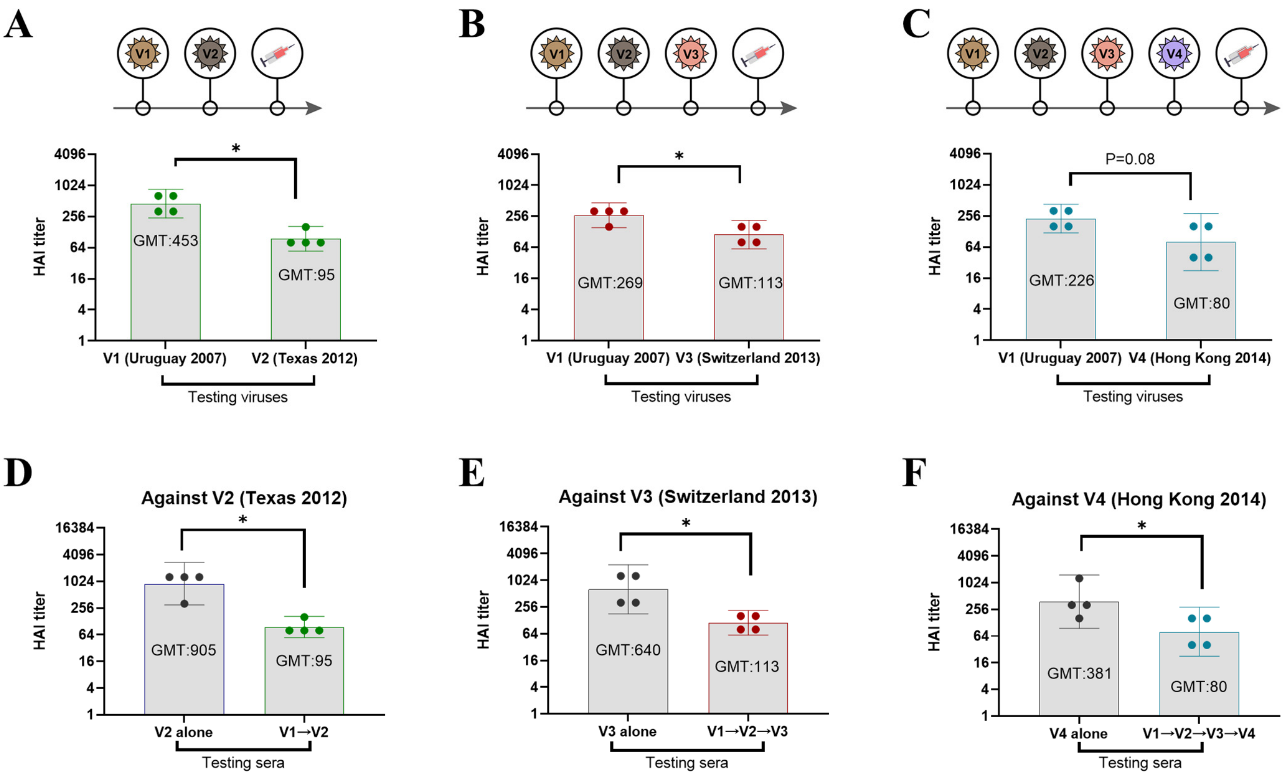

Figure S1. Hemagglutination inhibition (HAI) responses in ferrets with sequential H3N2 infections. Seronegative ferrets were sequentially infected by V_1=Uruguay 2007 followed by V_2=Texas 2012, then V_3=Switzerland 2013, and lastly V_4=Hong Kong 2014. Sera from each additional infection were tested for HAI titers against V1, V2, V3 or V4 as compared to those from ferrets singly infected with V2, V3 or V4. Bar graphs with error bars indicate geometric means (GMT, values shown) of HAI titers from four ferrets (points) per group with 95% confidential intervals. * indicates p<0.05 by Mann-Whitney test after log-transformation.

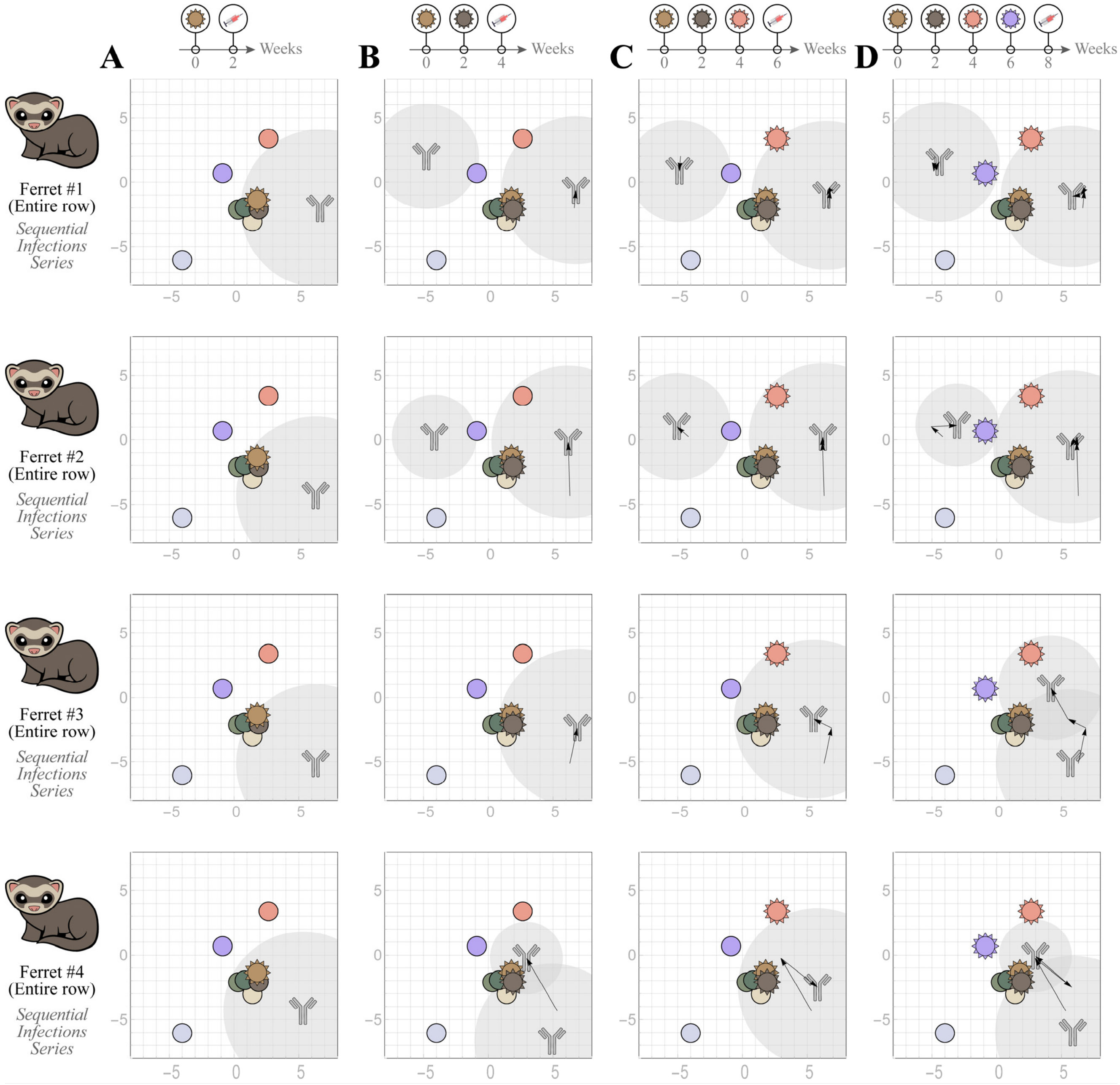

Figure S2. Tracing individual ferret responses through four sequential infections. Neutralization landscapes created using HAI titers from individual ferrets infected by (A) V_1=Uruguay 2007 followed by (B) V_2=Texas 2012, (C) V_3=Switzerland 2013, and lastly (D) V_4=Hong Kong 2014. Arrows track the motion of each antibody signature. The average response in Figure 1 depicts the geometric mean of the HAI titers from each column.

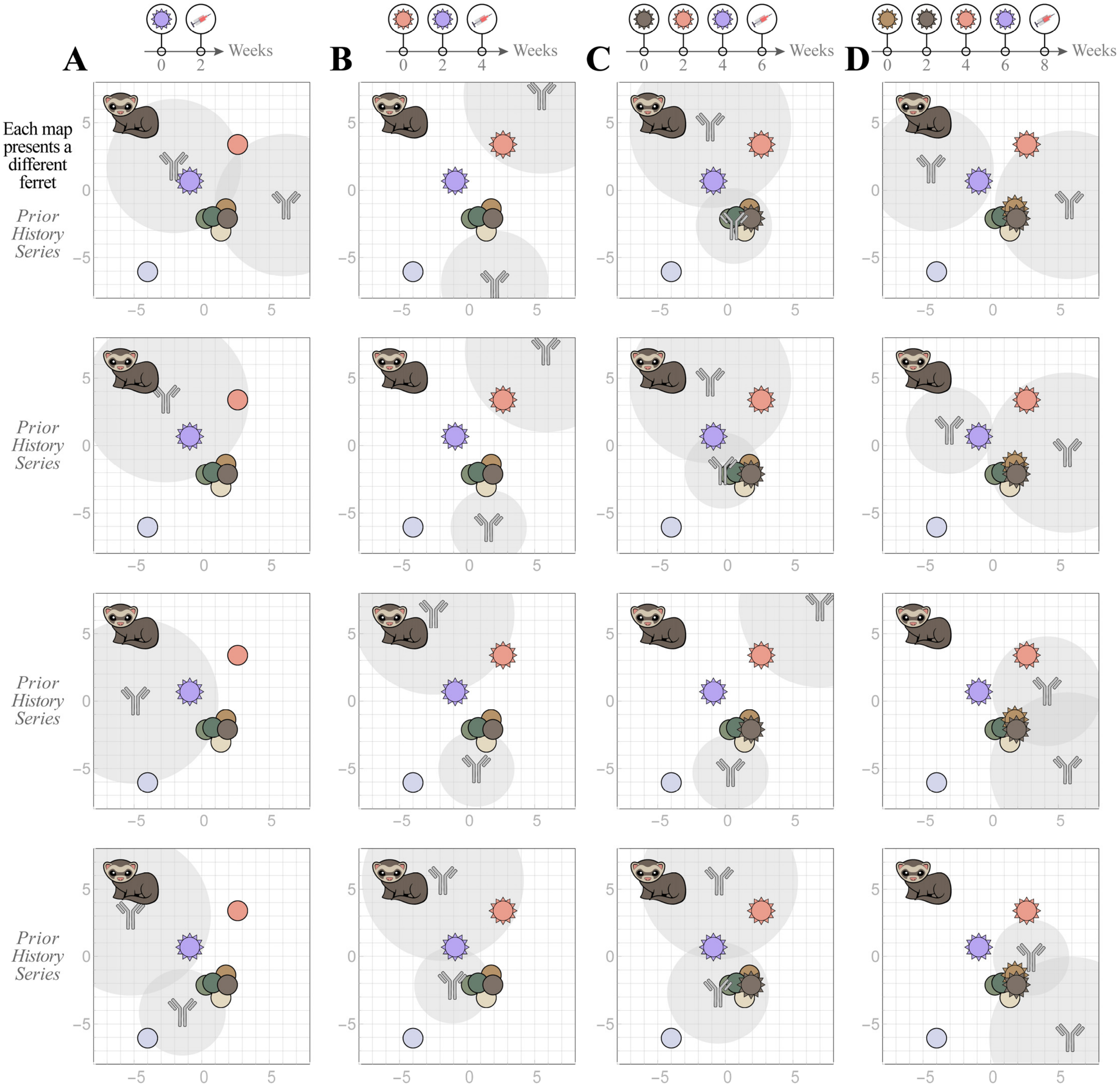

Figure S3. Tracing individual ferret responses with four different prior infection histories. Neutralization landscapes created using HAI titers from individual ferrets infected by (A) V_4 alone, (B) $V_3 \rightarrow V_4$, (C) $V_2 \rightarrow V_3 \rightarrow V_4$, or (D) $V_1 \rightarrow V_2 \rightarrow V_3 \rightarrow V_4$. A total of sixteen ferrets were tracked. The average response in Figure 2 depicts the geometric mean of the HAI titers from each column.

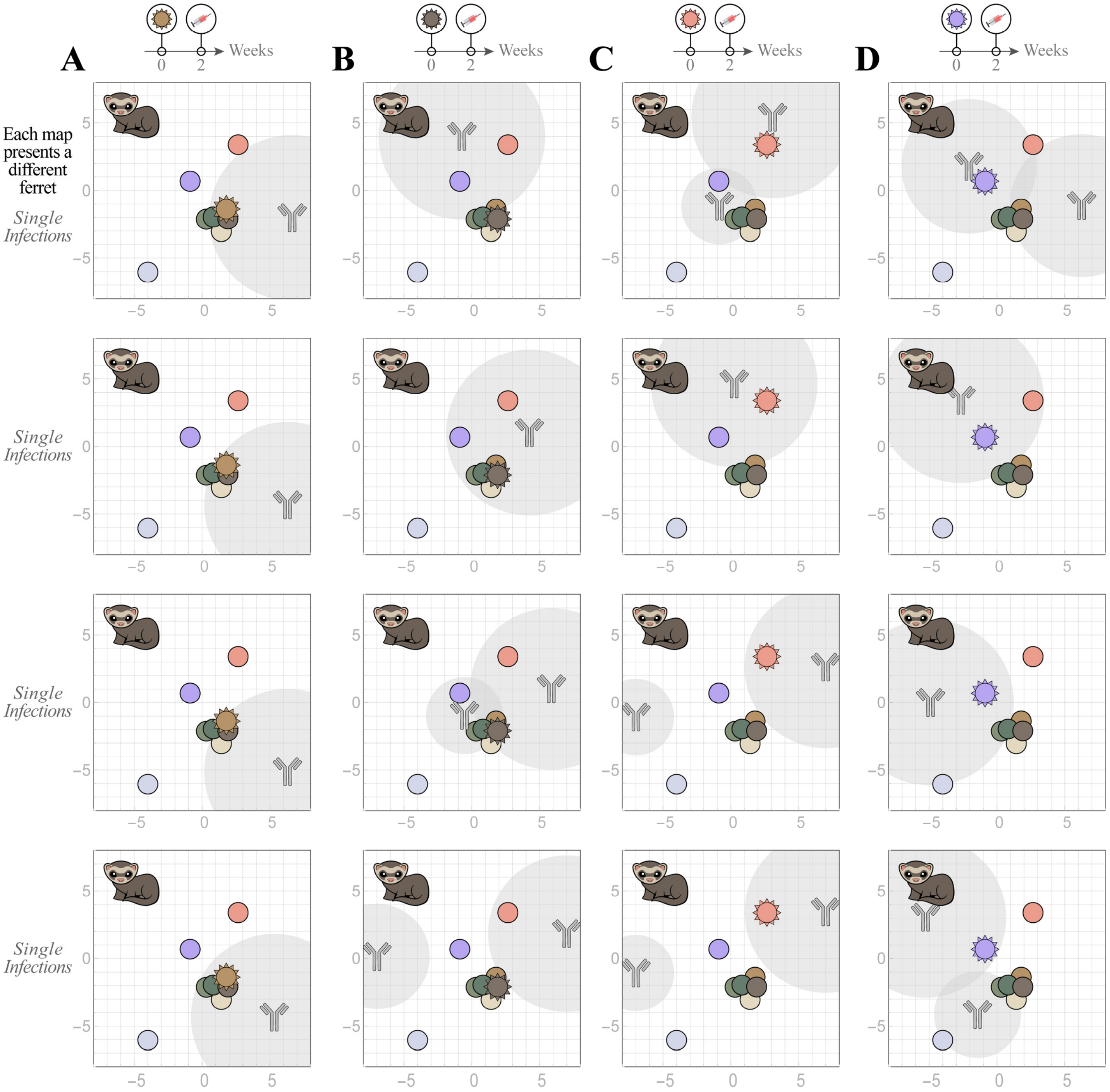

Figure S4. Antibody repertoires from single infections of ferrets. Neutralization landscapes created using HAI titers from individual ferrets infected by either (A) V_1, (B) V_2, (C) V_3, or (D) V_4 alone.

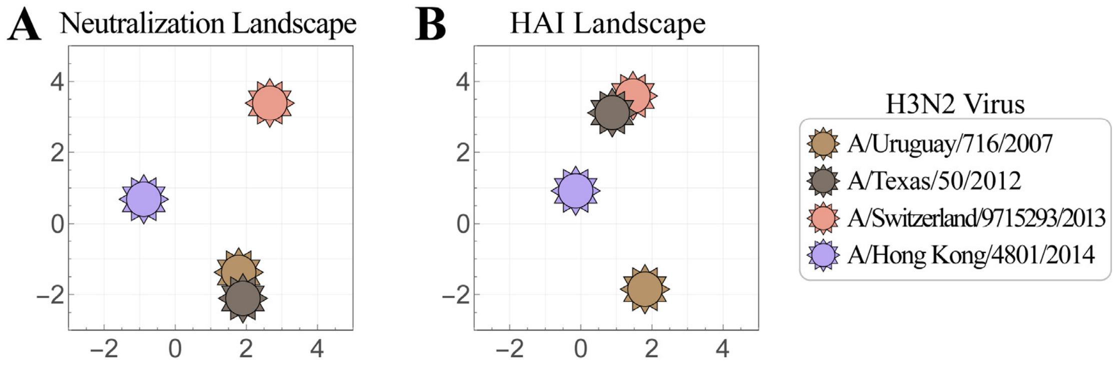

Figure S5. Comparing neutralization and HAI virus landscapes. (A) The positions of the four infecting strains derived in this manuscript using neutralization data from human monoclonal antibodies. (B) These same virus coordinates derived using HAI titers of ferret sera elicited by single infections (Figures 3A, 4A, 4B, 5A), using antigenic cartography. The position of A/Texas/50/2012 substantially shifts between panels.
